# [Retracted] Tunicamycin inhibits colon carcinoma growth and aggressiveness via modulation of the ERK‑JNK‑mediated AKT/mTOR signaling pathway

**DOI:** 10.3892/mmr.2024.13184

**Published:** 2024-02-19

**Authors:** Shuping You, Weihong Li, Yun Guan

Mol Med Rep 17: 4203–4212, 2018; DOI: 10.3892/mmr.2018.8444

Following the publication of this paper, it was drawn to the Editor's attention by a concerned reader that certain of the Transwell invasion and migration assay data shown in Fig. 1B and C were strikingly similar to data appearing in different form in other articles written by different authors at different research institutes, which had either already been published or were under consideration for publication at around the same time. Owing to the fact that the contentious data in the above article had already been published prior to its submission to *Molecular Medicine Reports*, the Editor has decided that this paper should be retracted from the Journal. The authors were asked for an explanation to account for these concerns, but the Editorial Office did not receive a reply. The Editor apologizes to the readership for any inconvenience caused.

